# Wide-Color-Span: Blue-Shifted Thermochromic D-A-D Fluorophores for Ratiometric Temperature Sensing

**DOI:** 10.3390/molecules31193504

**Published:** 2026-10-01

**Authors:** Zheng-Kun Qin, Chi Feng, Meiqi Tang, Yanfei Li, Qingao Zhang, Ruipeng Deng, Ming-Xing Song

**Affiliations:** 1 College of Information Technology, Jilin Engineering Research Center of Optoelectronic Materials and Devices, Jilin Normal University, Siping 136000, China; 2 Jilin Provincial Key Laboratory of Wide Bandgap Semiconductor Material Growth and Device Applications, Jilin Normal University, Changchun 130103, China; 3 State Key Laboratory of Rare Earth Resource Utilization, Changchun Institute of Applied Chemistry, Chinese Academy of Sciences, Changchun 130022, China

**Keywords:** D-A-D type molecules, thermochromic fluorescence, organic synthesis of optoelectronic materials, temperature sensing

## Abstract

Thermochromic fluorescent materials have significant application prospects in the field of temperature sensing. In this study, based on the donor–acceptor–donor (D-A-D) strategy, two fluorescent molecules, MPT-1 and MPT-2, with fluorenyl cores and methoxy phenothiazine as donors, were synthesized via the Buchwald–Hartwig C–N cross-coupling reaction, and their structures were confirmed by ^1^H NMR, ^13^C NMR and HRMS. Thermogravimetric analysis indicated that both compounds exhibited excellent thermal stability (T_d_ = 377 °C, 413 °C). Aggregation-induced emission (AIE) experiments showed that the emission peak blue-shifted by approximately 42–44 nm with increasing aggregation. Temperature-dependent fluorescence studies indicated that from 80 K to 300 K, the emission peaks of both molecules shifted continuously from the yellow region to the blue region. The relative sensitivities of MPT-1 and MPT-2 were 2.5% K^−1^ and 2.19% K^−1^, respectively, with CIE ΔE shifts of 0.19 and 0.18. This study elucidates the thermochromic mechanism of D-A-D-type fluorescent molecules, confirms the tunability of their emission colour in response to temperature, and provides theoretical guidance and experimental reference for the design of novel thermochromic fluorescent materials and their applications in temperature sensing.

## 1. Introduction

Stimulus-responsive luminescent materials can generate reversible optical feedback in response to external stimuli such as light, heat, and chemical environments, enabling the controlled modulation of parameters such as light colour, intensity, and lifetime [1,2,3]. They have demonstrated significant application value in fields such as smart displays and temperature sensing [4,5,6]. Among these, thermochromic luminescent materials have attracted widespread attention due to their advantages, including non-contact operation and real-time visualization [6,7,8]. However, existing organic thermochromic luminescent systems still face significant limitations in practical applications. The root cause of this bottleneck lies in the inherent contradiction of the thermochromic mechanism: an increase in temperature typically intensifies molecular thermal motion and activates non-radiative decay pathways, leading to structural relaxation of the excited state and a red shift in the emission peak [9,10]. To overcome this trend, new strategies must be introduced at the levels of molecular design and aggregation state regulation, such that an increase in temperature actually suppresses the charge transfer (CT) process and promotes emission dominated by localized excited (LE) states [11,12,13,14,15,16].

This requires the construction of a system at the molecular design level that can precisely regulate the energy competition between the CT state and the LE state. Donor–acceptor (D-A)-type conjugated systems, due to their inherent intramolecular charge transfer (ICT) characteristics, provide an ideal molecular platform for this regulation [17,18,19,20,21,22,23,24,25]. In recent years, research on the synergistic effects of aggregation-induced emission (AIE) and twisted intramolecular charge transfer (TICT) has further demonstrated that by controlling the intramolecular rotational freedom and aggregate-state structure, the non-radiative relaxation of the TICT state can be effectively suppressed, thereby directing the excited-state energy towards the LE-state radiative channel. The synergistic effect of AIE and TICT offers rich regulatory means for the construction of materials exhibiting abnormal thermally responsive luminescence [26,27,28,29,30,31,32,33,34].

Based on this, this study uses 2,7-dibromo-9,9-dimethylfluorene and 2,7-dibromo-9,9-diphenylfluorene as acceptors, and methoxyphenothiazine as a donor, synthesizing two D-A-D-type fluorescent molecules, MPT-1 and MPT-2, through the Buchwald–Hartwig C–N coupling. The study systematically investigates their thermal stability, photophysical properties, AIE behaviour, and temperature-dependent fluorescence response, revealing that the LE-CT competition model is the dominant mechanism in thermochromic behaviour. The research finds that MPT-1 and MPT-2 exhibit a significant continuous blue-shifted emission from yellow to blue light over the temperature range of 80–300 K, with a chromaticity shift covering nearly half of the visible spectrum, originating from temperature-induced TICT quenching and a transition of the emission mechanism from the CT state to the LE state. This study elucidates the structure–performance relationship and thermochromic mechanism of D-A-D molecules, providing new insights for the design of high-performance temperature-sensing materials.

## 2. Results and Discussion

### 2.1. Thermal Stability

**Figure 1:**
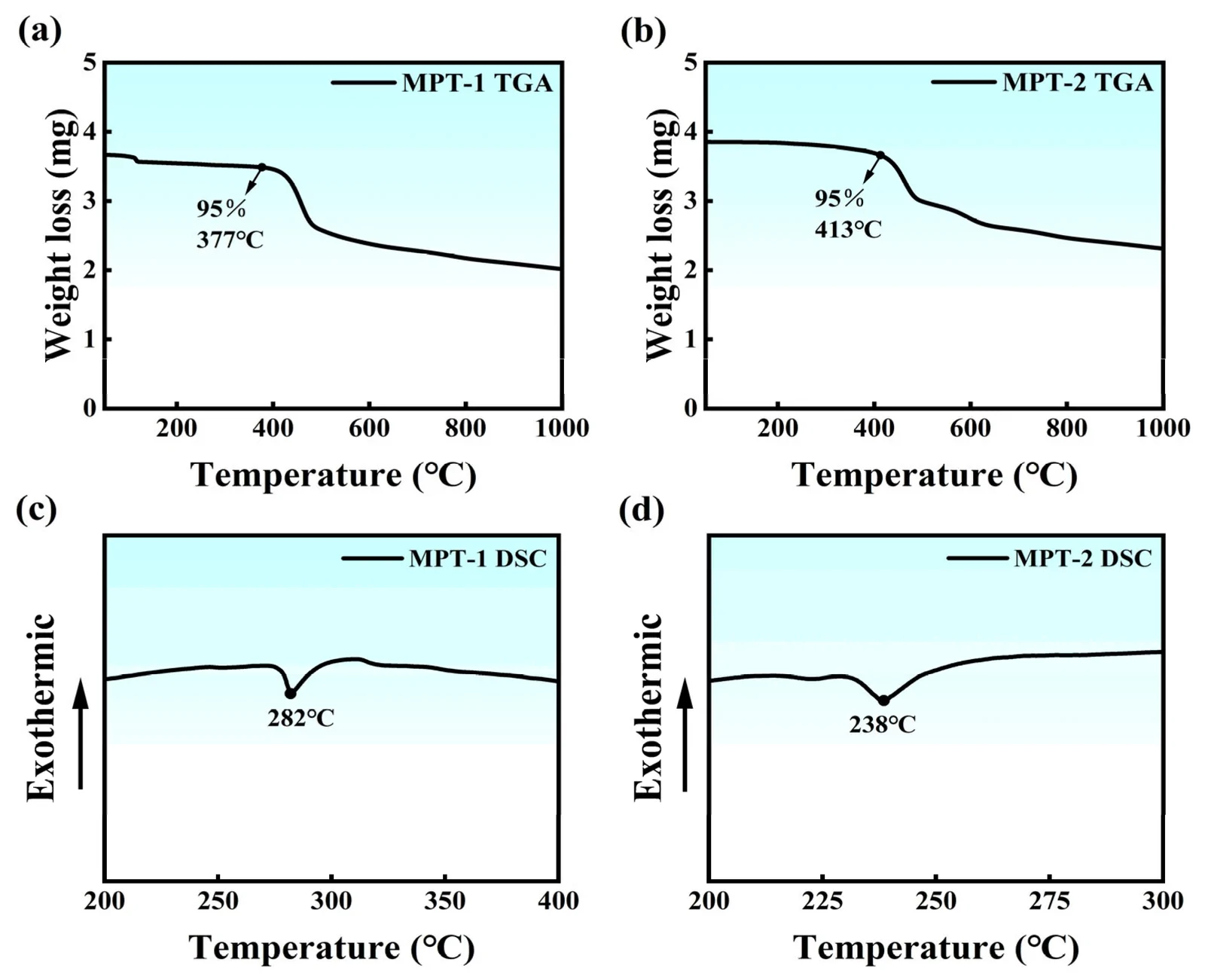
(**a**,**b**) correspond to the TGA curves of MPT-1 and MPT-2 respectively. (**c**,**d**) correspond to the DSC curves of MPT-1 and MPT-2 respectively. TG-DSC tests were carried out on a simultaneous thermal analyser (TGA/HT DSC, HSS2 sensor). The sample was placed in a 70 μL alumina ceramic crucible and heated from 30 to 1000 °C at 10 °C/min under a N_2_ atmosphere, Mass changes were recorded using a UMX5 microbalance(Mettler-Toledo GmbH, Greifensee, Switzerland). The instrument was calibrated for temperature and energy using an indium standard.

The thermal stability of MPT-1 and MPT-2 under a nitrogen atmosphere was investigated using thermogravimetric analysis (TGA) and differential scanning calorimetry (DSC) as shown in Figure 1. Both compounds exhibit excellent thermal stability: TGA results show that the 5% weight loss temperatures of MPT-1 and MPT-2 are 377 and 413 °C, respectively. In the DSC curves, MPT-1 and MPT-2 display distinct endothermic peaks at 282 and 238 °C, respectively. As there is no significant mass loss in the TGA curves at the corresponding temperatures and these temperatures are far below their decomposition temperatures, these endothermic peaks can be attributed to melting transitions rather than thermal decomposition. Collectively, these results indicate that both compounds effectively resist thermal degradation at high temperatures and maintain a high degree of structural integrity of their molecular frameworks under heat excitation, fully demonstrating the materials’ exceptional thermal performance and broad potential for high-temperature applications.

### 2.2. Spectral and Transient Fluorescence Lifetime Analysis

**Figure 2:**
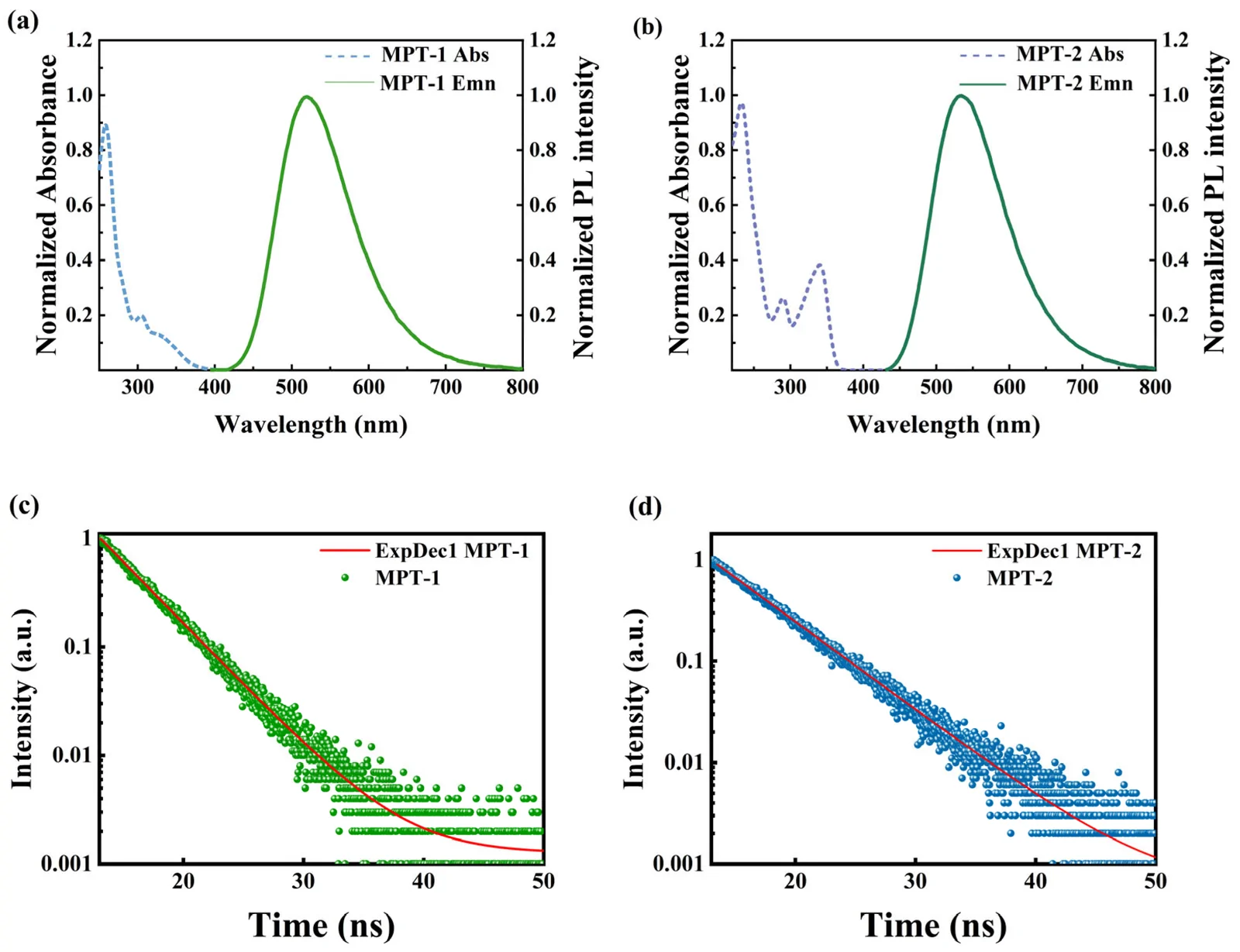
(**a**,**b**) show the UV–visible absorption (dashed lines) and fluorescence emission (solid lines) of MPT-1 and MPT-2. (**c**,**d**) show the Time-Correlated Single Photon Counting (TCSPC) decay curves of MPT-1 and MPT-2, respectively, fitted using a single-exponential model. The ultraviolet–visible absorption spectrum and fluorescence emission spectrum were measured at room temperature using a Shimadzu UV-2600i (Shimadzu Corporation, Kyoto, Japan, slit 3 nm) and a Shimadzu RF-6000 (Shimadzu Corporation, Kyoto, Japan, excitation/emission slit 5 nm), respectively, with an excitation wavelength of 370 nm, using dimethyl sulfoxide (DMSO) as the solvent and a sample concentration of 0.1 mg/mL.

Figure 2a,b show the UV–visible absorption spectra of MPT-1 and MPT-2, respectively; as shown, MPT-1 and MPT-2 display characteristic absorption peaks around 260 and 247 nm, respectively. TD-DFT/CAM-B3LYP/6-31G* calculations indicate that the strongest absorption peak of MPT-1 is located at 253 nm (4.8988 eV, f = 2.3030); the strongest transition of MPT-2 occurs at 253.09 nm (4.8989 eV, f = 2.5096). The above computational results are highly consistent with the experimental measurements.

MPT-1 and MPT-2 show typical green light emission (Table 1). Both compounds exhibit large Stokes shifts of 185 and 188 nm, respectively (as shown in Section S4), suggesting pronounced intramolecular charge transfer and structural relaxation in their excited states, consistent with literature reports on analogous compounds [35,36,37,38]. Fitting the data to a single-exponential model in Figure 2c,d yielded a lifetime of τ_1_ ≈ 3.80 ns for MPT-1 and a lifetime of τ_2_ ≈ 4.92 ns for MPT-2, with the residual distribution meeting statistical requirements (as shown in Figure S2). These lifetimes fall within the typical nanosecond range for organic fluorescence, ruling out the possibility of long-lived phosphorescence and being consistent with the expected rapid radiative decay of allowed transitions in the D-A system. Additionally, according to Equations (1) and (2),

(1) $$k_{r}=\frac{\Phi }{\tau }$$

(2) $$k_{nr}=\frac{1-\Phi }{\tau }$$

the radiative rate constant k_r_ and non-radiative rate constant k_nr_ were calculated for both solution and solid states, and the results are summarized in Table 2.

The k_nr_ values of MPT-1 and MPT-2 are more than an order of magnitude higher than k_r_, indicating the dominant role of non-radiative decay channels in the deactivation of the excited state. This non-radiative dominance mainly arises from twisted intramolecular charge transfer (TICT) of D-A molecules in the excited state in solution, accompanied by intramolecular rotational and vibrational relaxation. The D-A structure inherently possesses strong CT characteristics, making the excited state prone to donor–acceptor conformational twisting. Simultaneously, intramolecular rotational and vibrational relaxation provides additional non-radiative dissipation channels; these channels significantly increase k_nr_, resulting in a notable difference in magnitude between the two. This result is consistent with the relatively low PLQY in solution (Table 1).

### 2.3. Solvatochromic Effect

**Figure 3:**
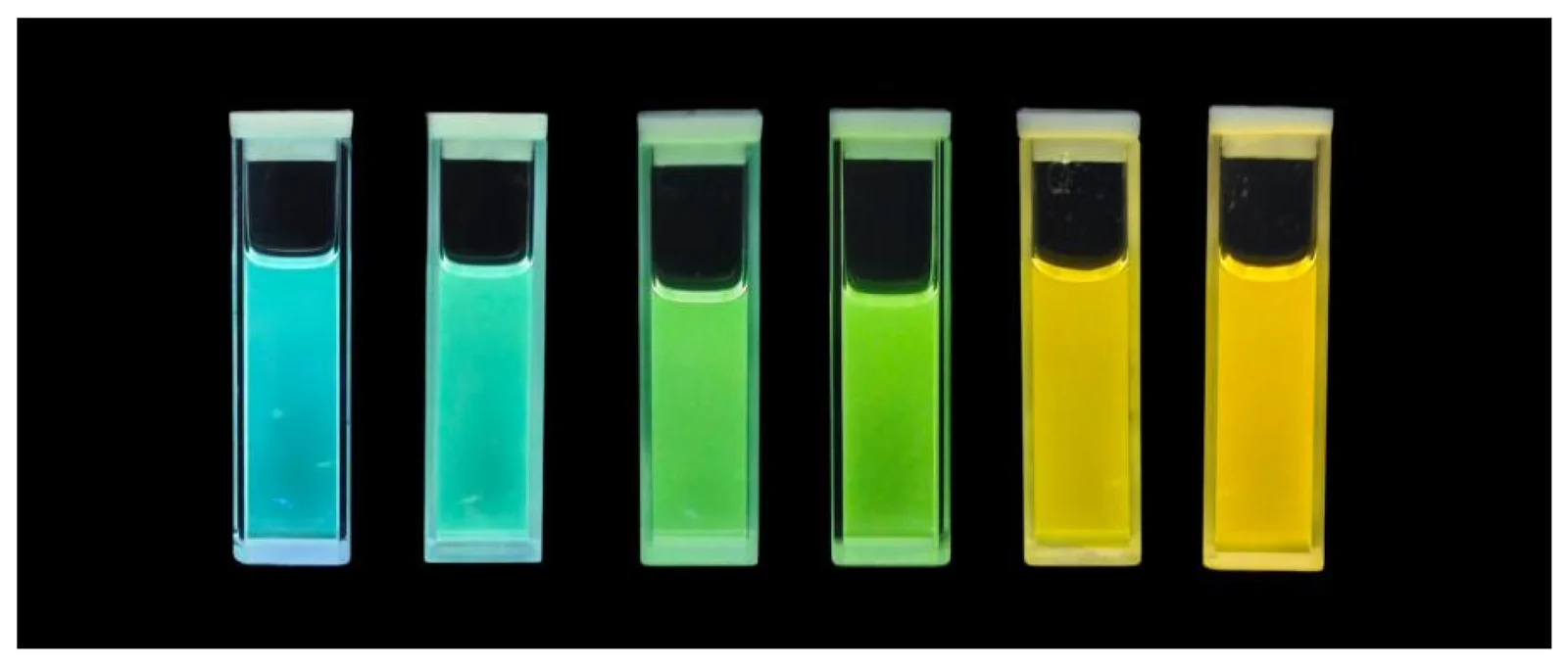
Samples are numbered 1–6 from left to right: 1 and 2 represent blue light (MPT-1/MPT-2, n-hexane), 3 and 4 represent green light (MPT-1/MPT-2, dichloromethane), and 5 and 6 represent yellow light (MPT-1/MPT-2, DMSO).

To gain a deeper understanding of the charge transfer characteristics of this system, we examined the solvent-induced colour effects of MPT-1 and MPT-2 in n-hexane, dichloromethane, and DMSO in Figure 3. As the solvent polarity increased from hexane to DMSO, the emission bands of both compounds exhibited significant red shifts in Table 3. This result indicates that the environmental polarity can significantly regulate the energy of the CT state, providing an important basis for understanding the subsequent temperature-dependent energy regulation behaviour.

### 2.4. Temperature-Dependent Solid-State Fluorescence Spectroscopy Analysis and Colour Change upon Heating

**Figure 4:**
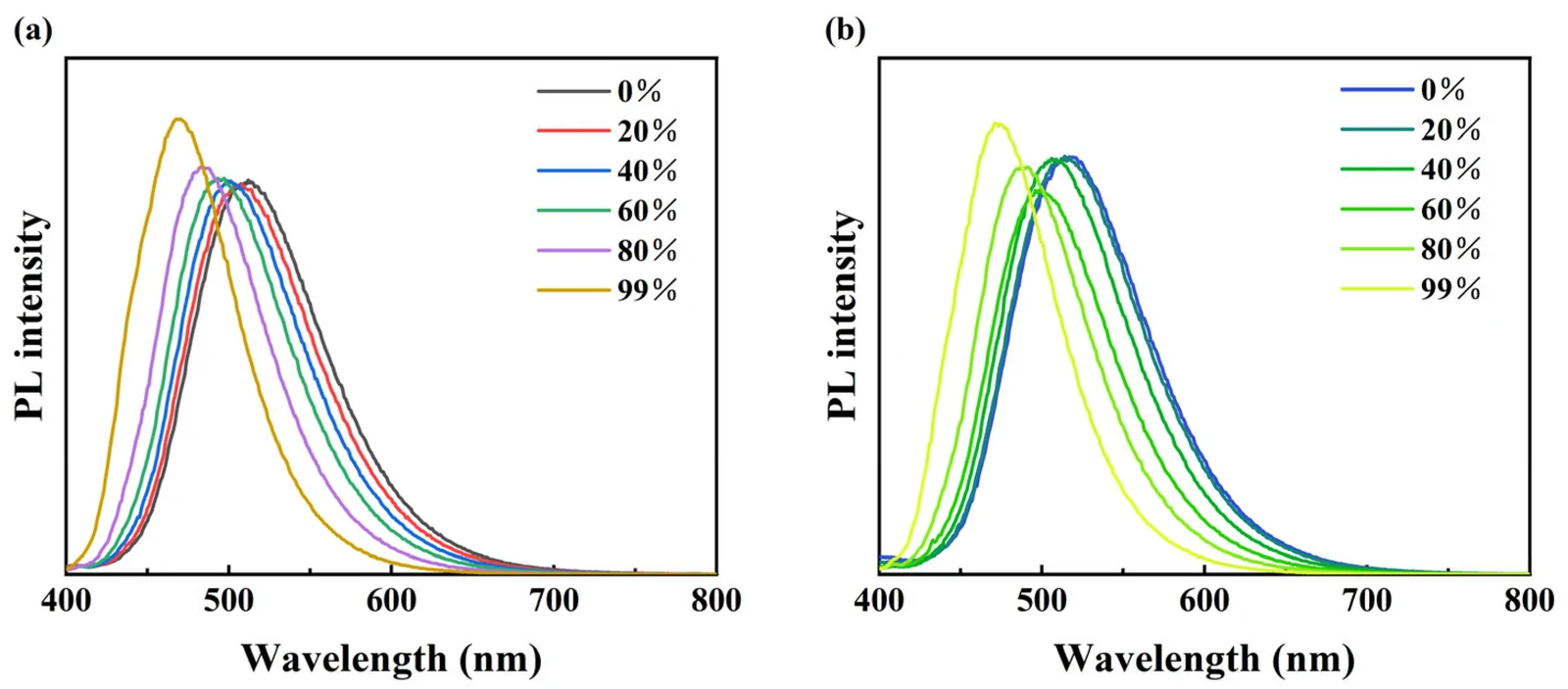
Fluorescence spectra of MPT-1 (**a**) and MPT-2 (**b**) in DCM/n-hexane mixed solutions with different n-hexane volume fractions (f_hex_). Testing conditions: concentration—0.1 mg/mL, excitation wavelength—365 nm, slit—3 nm.

AIE titration results show that, as the n-hexane volume fraction increases, the emission peaks of MPT-1 and MPT-2 blue-shift by 42 and 44 nm, respectively (Table 4), and the fluorescence intensity is enhanced (Figure 4a,b). This is attributed to the restriction of intramolecular motion in the aggregated state, effectively suppressing non-radiative relaxation of the twisted intramolecular charge transfer (TICT) state, making high-energy locally excited (LE)-state emission dominant.

Temperature-dependent fluorescence experiments further support this inference. In the solid state, as the temperature rises from 80 K to 300 K, the fluorescence intensity continuously decreases, with a slight red shift in the emission peak (Table 5). This thermal quenching behaviour is attributed to heating, which intensifies the molecular thermal vibrations, accelerates the non-radiative processes of the TICT state, and selectively quenches the CT component. The high-energy LE peak remains nearly unchanged at high temperatures, suggesting that the LE state possesses a more rigid structure and is less affected by thermal perturbation, during excited-state relaxation. Radiative transitions (LE state) and non-radiative transitions (CT state) compete. Spatial constraints in the aggregated state direct excitons towards the LE emission pathway (blue shift), whereas thermal energy perturbation upon heating promotes the TICT non-radiative pathway (quenching, reduction in the CT component).

**Figure 5:**
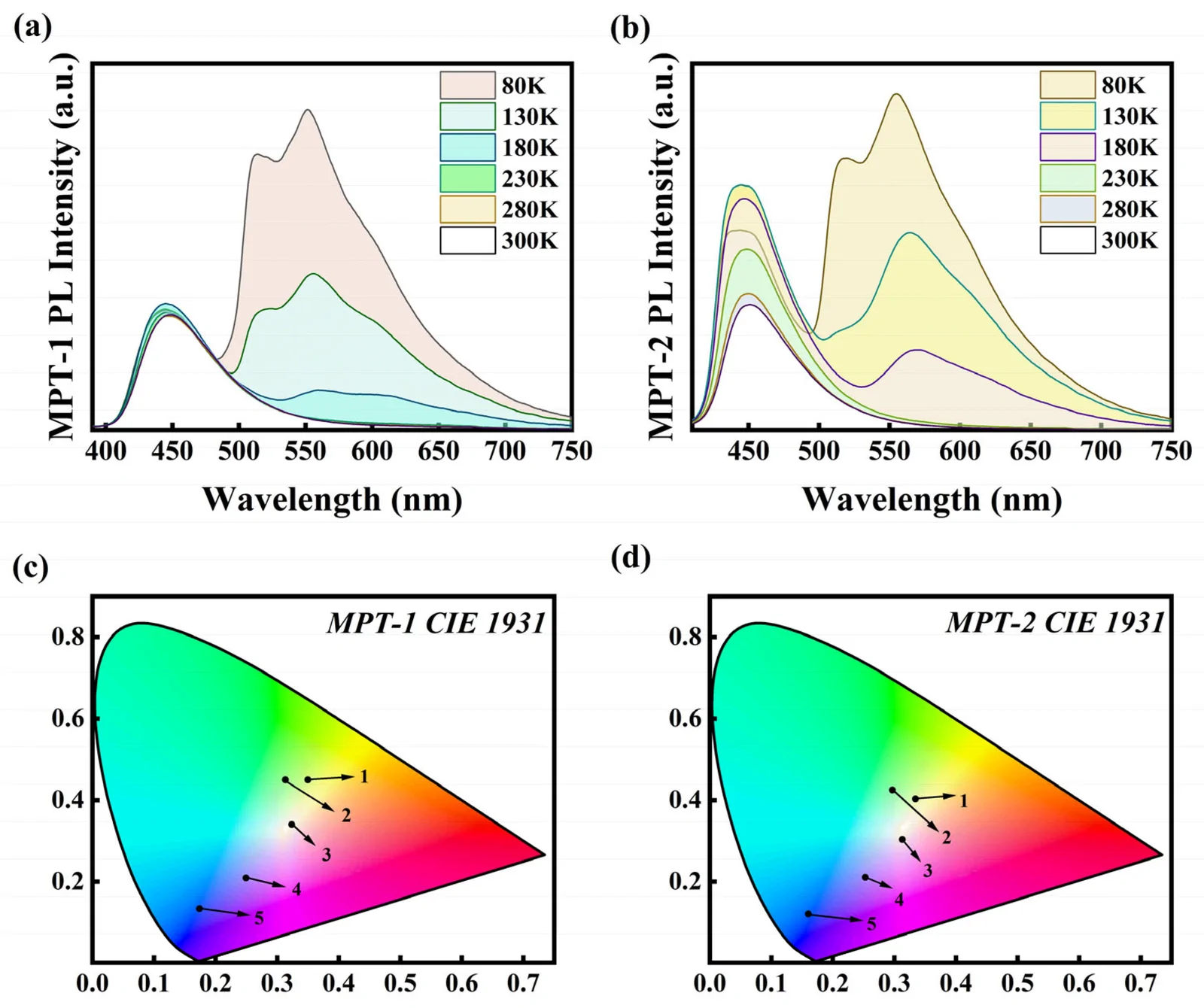
Temperature-dependent solid-state photoluminescence spectra (**a**,**b**) and corresponding chromatic trajectories (**c**,**d**) for MPT-1 and MPT-2 in the visible region. Data points numbered 1–5 correspond to 80 K, 130 K, 180 K, 230 K and 280 K, respectively, with the numbering sequence indicating the direction of heating (from 1 to 5).

Both aspects collectively confirm that the freedom of intramolecular motion is the core regulatory switch of the emission colour and intensity in this system. The essence of the thermochromic behaviour lies in the significant reversal of the dual emission intensity ratio (LE/CT) show in Figure 5a,b, which is reflected in Figure 5c,d as a continuous shift in the CIE chromaticity coordinates from the yellow-green region to the blue region (specific coordinates listed in Table 6), achieving a visually unique blue-shifted thermochromic phenomenon. This phenomenon is consistent with reports on thermochromic materials in the literature [39,40,41].

### 2.5. Temperature-Sensing Performance

#### 2.5.1. FIR Definition and Thermal Response

The temperature-dependent PL spectra of MPT-1 and MPT-2 in Figure 5a,b exhibit a distinct dual-band emission profile. The high-energy band centred at 450 nm originates from a localized excited state and remains nearly invariant with temperature, serving as a robust built-in reference signal (I_LE_). In contrast, the low-energy band centred at 520 nm and 533 nm, assigned to an emissive state susceptible to thermally activated non-radiative decay, undergoes pronounced thermal quenching and thus, based on the proportional temperature measurement principle, functions as the temperature-sensitive signal (I_CT_). The fluorescence intensity ratio (FIR) is defined as [42,43,44]

(3) $$FIR=\frac{I_{CT}}{I_{\mathrm{LE}}}$$

where and $I_{\mathrm{LE}}$ and $I_{\mathrm{CT}}$ denote the integrated PL intensities over 420–470 nm and 500–650 nm, respectively. The extracted FIR values are compiled in Table 7.

As depicted in Table 7, the FIR values of both compounds decrease monotonically with increasing temperature. This trend reflects the gradual thermal activation of non-radiative decay channels of the low-energy CT emissive state, resulting in selective quenching of CT emission during heating, while the high-energy LE emission remains almost unchanged, which is consistent with the observed thermally induced colour shift and the ratiometric temperature-sensing mechanism.

#### 2.5.2. Sensitivity Analysis

The temperature-sensing capability is quantified by two complementary parameters, absolute sensitivity (S_a_) and relative sensitivity (S_r_):

(4) $$S_{r}=\frac{1}{\mathrm{FIR}}\cdot \frac{d(\mathrm{FIR})}{d(T)}\times 100\%=\frac{d(\ln\;\mathrm{FIR})}{d(T)}\times 100\%$$

(5) $$S_{a}=\;|\frac{d(\mathrm{FIR})}{d(T)}|$$

To avoid bias from model assumptions, the derivatives were computed numerically using the central difference method for interior data points, with forward and backward differences applied at the boundaries:

(6) $$\frac{d(\mathrm{FIR})}{d(T)}{|}_{Ti}\approx \frac{{\mathrm{FIR}}_{i+1}-{\mathrm{FIR}}_{i-1}}{T_{i+1}-T_{i-1}}\;\;\;\;\;\;(i=2,3,\ldots ,n-1)$$

The calculated sensitivity values are summarized in Table 8.

The temperature dependence of S_r_ exhibits non-monotonic behaviour that can be rationalized by the interplay between the FIR magnitude and its temperature derivative. In the low-temperature regime (80–180 K), S_r_ increases gradually despite a decrease in d(FIR)/d(T), because the concurrent reduction in FIR partially compensates for the diminished slope. This yields local maxima of 2.50% K^−1^ (MPT-1) and 2.19% K^−1^ (MPT-2) at 180 K. Between 180 K and 230 K, S_r_ declines because the FIR denominator decreases more slowly than d(FIR)/d(T), suppressing the normalized sensitivity.

The absolute sensitivity S_a_ decreases monotonically from 80 K to 230 K, reflecting the progressively saturated thermal quenching rate as the emissive state population is depleted. Beyond 230 K, S_a_ plateaus at a low level, consistent with the near-complete depopulation of the thermally quenched state.

#### 2.5.3. Chromaticity Analysis

The macroscopic colour evolution was quantified via CIE 1931 chromaticity coordinates (Table 6) extracted from the temperature-dependent spectra. The Euclidean distance between two temperatures is defined as

(7) $$\Delta E=\sqrt{{\left(x_{2}-x_{1}\right)}^{2}+{\left(y_{2}-y_{1}\right)}^{2}}$$

and the colorimetric sensitivity as

(8) $$S_{\mathrm{color}}=\frac{\Delta E}{\Delta T}$$

Total chromatic shift (80 → 300 K): MPT-1: ΔE = 0.19, MPT-2: ΔE = 0.18. Average chromatic sensitivity: MPT-1: S_color_ = 0.86 × 10^−3^ K^−1^, MPT-2: S_color_ = 0.82 × 10^−3^ K^−1^.

## 3. Materials and Methods

### 3.1. Synthesis and Characterization

#### 3.1.1. Synthesis Route

**Figure 6:**
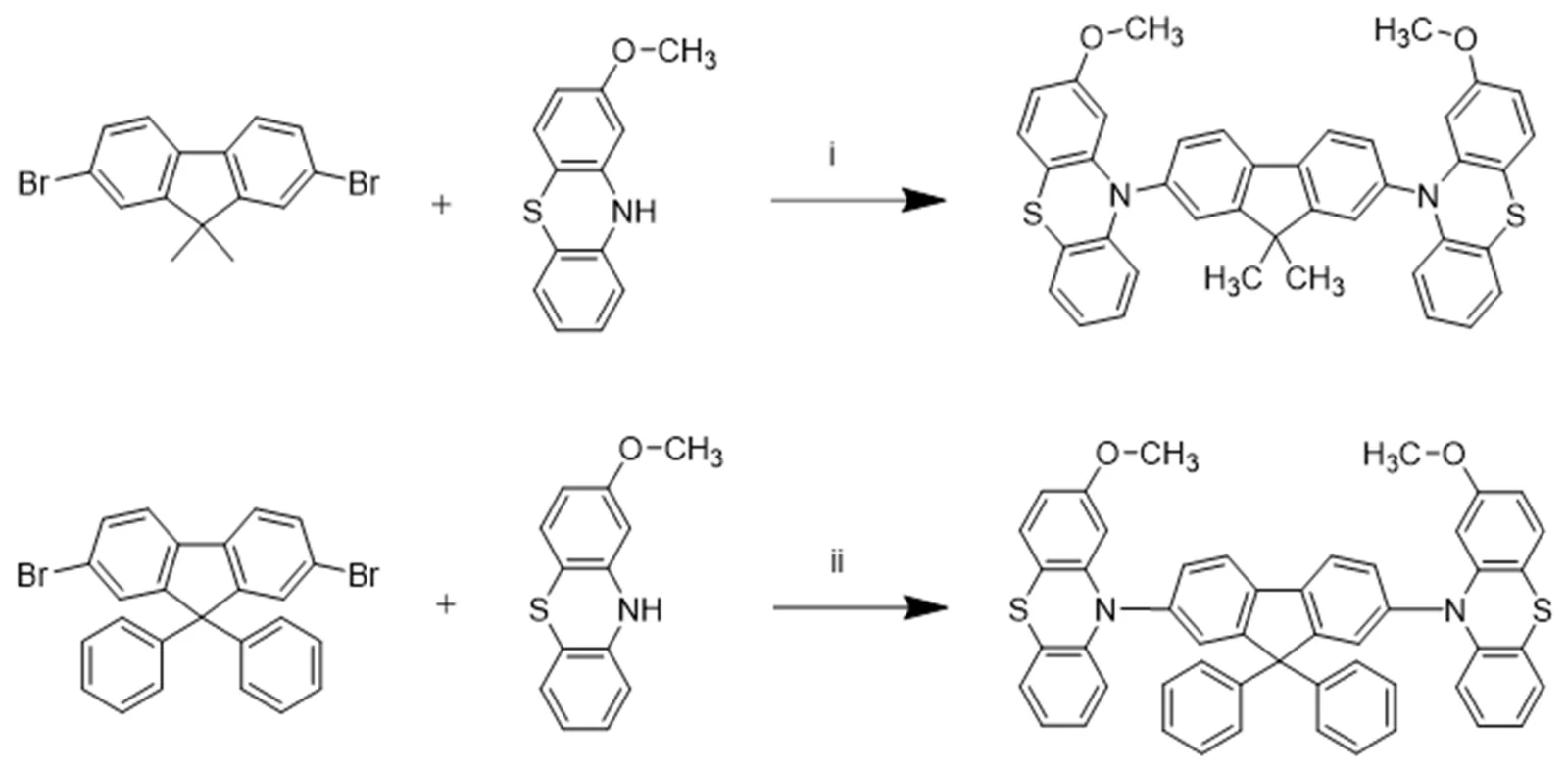
Synthesis routes for MPT-1 (i) and MPT-2 (ii). i,ii: The catalytic system consists of tri-tert-butylphosphine (P(t-Bu)_3_) and palladium acetate (Pd(OAc)_2_), with sodium tert-butoxide (NaOt-Bu) providing the basic environment and toluene as the solvent; the mixture is heated to 110 °C and reacted for 48 h.

This study employed 2,7-dibromo-9,9-dimethylfluorene and 2,7-dibromo-9,9-diphenylfluorene as starting materials, and methoxyphenothiazine as the amine coupling partner. The target compounds MPT-1 and MPT-2 were successfully synthesized via a palladium-catalyzed Buchwald–Hartwig C–N cross-coupling reaction, facilitated by a tri-tert-butylphosphine ligand [45], as illustrated in Figure 6. (MPT-1: 2,7-Bis(7-methoxy-10H-phenothiazin-10-yl)-9,9-dimethyl-9H-fluorene(C_41_H_32_N_2_O_2_S_2_), MPT-2: 2,7-Bis(7-methoxy-10H-phenothiazin-10-yl)-9,9-diphenyl-9H-fluorene(C_51_H_36_N_2_O_2_S_2_)).

#### 3.1.2. Characterization Data

The structures were fully characterized by ^1^H NMR, ^13^C NMR, and high-resolution mass spectrometry (HRMS); all data were in good agreement with the proposed structures (as shown in Figures S3–S8).

MPT-1: ^1^H NMR (400 MHz, DMSO) δ 8.23 (d, J = 8.0 Hz, 1H), 7.75 (d, J = 1.9 Hz, 1H), 7.46 (dd, J = 8.0, 1.9 Hz, 1H), 7.09 (s, 1H), 7.02 (d, J = 8.4 Hz, 1H), 6.94 (t, J = 7.7 Hz, 1H), 6.87 (t, J = 7.4 Hz, 1H), 6.52 (dd, J = 8.5, 2.5 Hz, 1H), 6.24 (d, J = 8.1 Hz, 1H), 5.82 (d, J = 2.6 Hz, 1H), 3.53 (s, 3H), 1.55 (s, 3H). ^13^C NMR (101 MHz, DMSO) δ 159.35, 157.48, 145.50, 144.07, 140.04, 138.34, 130.22, 129.38, 128.69, 127.69, 127.66, 127.08, 125.80, 123.37, 123.14, 120.15, 116.35, 110.25, 107.64, 103.81, 55.47, 47.82, 31.44, 26.77, 22.55, 14.46. HRMS (ESI): m/z calcd for C_41_H_33_N_2_O_2_S_2_^+^ [M + H]^+^ 649.1978, found 649.1956. R_f_ = 0.61 (silica gel, n-Hex/EtOAc = 5:1).

MPT-2: ^1^H NMR (400 MHz, DMSO) δ 8.34 (d, J = 8.0 Hz, 1H), 7.53 (dd, J = 8.0, 1.9 Hz, 1H), 7.50 (d, J = 1.9 Hz, 1H), 7.24 (m, 5H), 7.07 (dd, J = 7.3, 1.9 Hz, 1H), 6.98 (d, J = 8.4 Hz, 1H), 6.88 (m, 2H), 6.47 (dd, J = 8.5, 2.5 Hz, 1H), 6.18 (dd, J = 7.9, 1.5 Hz, 1H), 5.72 (d, J = 2.5 Hz, 1H), 3.38 (s, 3H). ^13^C NMR (101 MHz, DMSO) δ 159.34, 154.37, 145.28, 144.99, 143.87, 140.54, 139.23, 130.63, 129.08, 128.61, 127.97, 127.78, 127.57, 127.17, 124.14, 123.33, 120.55, 116.45, 110.48, 108.69, 103.31, 65.74, 55.38. HRMS(ESI): m/z calcd for C5_1_H_36_NaN_2_O_2_S_2_^+^ [M + Na]^+^ 795.2110, found 795.2097. R_f_ = 0.57(silica gel, n-Hex/EtOAc = 5:1).

## 4. Conclusions

This work reports the design and synthesis of two fluorescent molecules, MPT-1 and MPT-2, based on a D-A-D framework via the Buchwald–Hartwig C–N cross-coupling reaction, with their structures confirmed by ^1^H NMR, ^13^C NMR and HRMS. In addition, the photophysical properties and thermochromic luminescence behaviour in the solid state were systematically investigated.

Experimental results showed that as the temperature increased from 80 K to 300 K, both compounds exhibited significant and continuous abnormally blue-shifted fluorescence emission, with the emission peaks shifting from the yellow region to the blue region, and the chromaticity shift covering nearly half of the visible light spectrum. This phenomenon is attributed to temperature-induced aggregate dissociation and the transition of the emission mechanism from the CT state to the LE state, highlighting the crucial role of excited-state configuration regulation in thermochromism. In terms of temperature-sensing performance, MPT-1 and MPT-2 possess relatively excellent sensitivity and chromatic response performance, with relative sensitivities of 2.5% K^−1^ and 2.19% K^−1^, and CIE chromaticity shifts ΔE of 0.19 and 0.18, respectively.

In summary, this study elucidates the intrinsic structure–property relationship of such materials, confirming the controllability of their emission colour and temperature response, and demonstrates their promising potential for temperature sensing and broader optical applications.

## Figures and Tables

**Table 1:** Photophysical properties of compounds MPT-1 and MPT-2.

Compounds	*λ_rem_* ^(a)^ (nm)	PLQY ^(b)^ (%)	PLQY ^(c)^ (%)	τ ^(d)^ (ns)
MPT-1	520	2.48	3.48	3.80 ± 0.010
MPT-2	533	4.66	5.97	4.92 ± 0.015

^(a)^: Measured emission main peaks at room temperature. ^(b)^: Photoluminescence quantum yield measured in solution (as shown in Supplementary Materials). ^(c)^: Photoluminescence quantum yield measured in the solid state (as shown in Supplementary Materials). ^(d)^: Fluorescence lifetimes of MPT-1 and MPT-2 in solution.

**Table 2:** Determined rate constants for compounds MPT-1 and MPT-2.

Compounds	k_r_ ^(a)^ (S^−1^)	k_nr_ ^(b)^ (S^−1^)
MPT-1	9.09 × 10^6^	2.52 × 10^8^
MPT-2	1.196 × 10^7^	1.884 × 10^8^

^(a)^: radiative rate constants of MPT-1 and MPT-2 in solution. ^(b)^: non-radiative rate constants of MPT-1 and MPT-2 in solution.

**Table 3:** The emission wavelengths of MPT-1 and MPT-2 are affected by the polarity of the solvent.

Compounds	*λ* ^(a)^ (nm)	*λ* ^(b)^ (nm)	*λ* ^(c)^ (nm)
MPT-1	471	511	544
MPT-2	473	516	550

^(a)^: Emission wavelength in n-hexane. ^(b)^: Emission wavelength in dichloromethane. ^(c)^: Emission wavelength in DMSO solution.

**Table 4:** AIE behaviour of MPT-1 and MPT-2 in DCM/n-hexane mixtures with different hexane fractions.

f_hex_ (%)	*λ* ^(a)^ (nm)	*λ* ^(b)^ (nm)
0	512	517
20	508	514
40	501	507
60	494	449
80	484	488
99	470	473

f_hex_: Volume fraction of n-hexane in the mixture. ^(a)^: Emission wavelength of MPT-1 at the corresponding volume fraction. ^(b)^: Emission wavelength of MPT-2 at the corresponding volume fraction.

**Table 5:** Emission peak data of MPT-1 and MPT-2 in the solid state at varying temperatures.

T(K)	*λ* ^(a)^ (nm)	*λ* ^(b)^ (nm)
80	444/553	445/555
130	446/556	445/565
180	445/560	447/570
230	446	449
280	447	450
300	448	451

^(a)^: The emission peak value of MPT-1. ^(b)^: The emission peak value of MPT-2.

**Table 6:** CIE chromaticity coordinates of MPT-1 and MPT-2.

T(K)	MPT-1 (x,y)	MPT-2 (x,y)
80	(0.35, 0.45)	(0.33, 0.40)
100	(0.31, 0.45)	(0.30, 0.42)
160	(0.32, 0.34)	(0.31, 0.30)
180	(0.25, 0.21)	(0.25, 0.21)
230	(0.17, 0.13)	(0.16, 0.13)

**Table 7:** FIR values of MPT-1 and MPT-2 at different temperatures.

T(K)	MPT-1	MPT-2
80	2.60	1.45
130	0.96	0.58
180	0.31	0.21
230	0.18	0.11

**Table 8:** Relative sensitivities of MPT-1 and MPT-2.

T(K)	MPT-1 S_r_ (% K^−1^)	MPT-1 S_a_ (K^−1^)	MPT-2 S_r_ (% K^−1^)	MPT-2 S_a_ (K^−1^)
80	1.262	0.0328	1.201	0.0174
130	2.385	0.0229	2.139	0.0124
180	2.516	0.0078	2.238	0.0047
230	1.444	0.0026	1.818	0.0020

## Data Availability

The data presented in this study are available on request from the corresponding author.

## Supplementary Materials

The following supporting information can be downloaded at https://www.mdpi.com/article/10.3390/molecules31193504/s1. Figure S1: Molecular structures and atomic numbering schemes for Compound 1 and Compound 2; Figure S2: The fitting results of TCSPC; Figures S3–S8: ^1^H NMR, ^13^C NMR and HR-MS spectrum of MPT-1 and MPT-2 in DMSO. Table S1: HOMO-LOMO energy level data.
