# Supplementary material for: Wide-Color-Span: Blue-Shifted Thermochromic D-A-D Fluorophores for Ratiometric Temperature Sensing
Source: Molecules. 2026 Oct 1;31(19):3504. doi: 10.3390/molecules31193504 (PMC13666673; doi:10.3390/molecules31193504)
Supplement: Supplementary file 1 [file molecules-31-03504-s001.zip › molecules-4585245-supplementary.pdf]

Supplementary Material

Wide-Color-Span Blue-Shifted Thermochromic D-A-D Fluorophores for Ratiometric Temperature Sensing

S1. Details of Material Synthesis and Purification

Synthesis of MPT-1: 2,7-dibromo-9,9-dimethylfluorene (1.0 mmol) and methoxyphenothiazine (2.2 mmol) were placed in a dry Schlenk tube; palladium acetate ($Pd(OAc)_2$, 0.3 mmol), tri-tert-butylphosphine ($P(t\text{-}Bu)_3$, 0.3 mmol ≈ 0.2 mL), and sodium tert-butylate *NaOt-Bu*,12*mmol* were then added. Anhydrous toluene (12 mL) was added, and the mixture was heated to 110 °C under nitrogen protection and allowed to react for 48 h. After the reaction, the mixture was cooled to room temperature. Thin-layer chromatography (TLC) was used to monitor the reaction progress and determine the eluent ratio. The product was purified by silica gel column chromatography using a n-hexane/ethyl acetate eluent (200:40 by volume). Trace impurities remained in the solution after column chromatography, so further purification was performed by recrystallization: the solution was refrigerated until a flocculent solid precipitated and then filtered through a sand-core funnel. The filter cake was washed with a small amount of solvent, rotary-evaporated under vacuum, transferred to a glass Petri dish, and dried in a vacuum oven at 120 °C, ultimately yielding a white powdery pure product.

Synthesis of MPT-2: The reaction conditions were essentially the same as those described above, with the starting material ratio adjusted to 2,7-dibromo-9,9-diphenylfluorene (1.0 mmol) and methoxyphenothiazine (2.3 mmol). During column chromatography, a pale yellow precipitate was observed in the eluent tube, attributed to the product's poor solubility and high synthesis yield. After column chromatography, rotary evaporation was used to remove the solvent, yielding a pale yellow solid. This was transferred to a glass Petri dish and dried under vacuum at 120 °C to obtain a pale yellow crystalline powder.

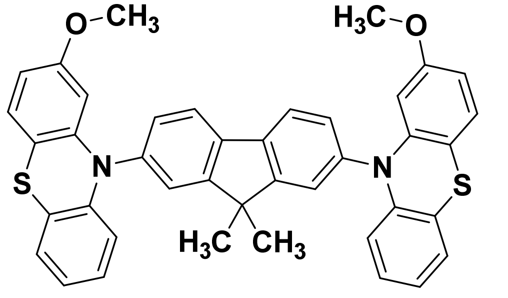

MPT-1

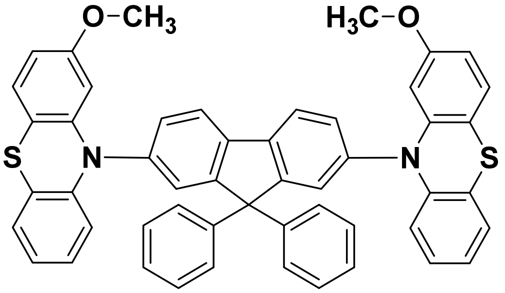

MPT-2

Figure S1. Molecular structures and atomic numbering schemes for Compound 1 and Compound 2.

S2. Conversion Relationship

The absorption wavelength can be obtained through the conversion of ΔE=LOMO-HOMO(energy differences). The formula is as follows:

$$\lambda(\text{nm})=\frac{1240}{E(ev)} \qquad S(1)$$

Table S1. HOMO-LOMO energy level data (1 a.u. = 27.2114 eV).

Compounds	HOMO(a.u.)	LUMO(a.u.)
MPT-1	-0.17692	-0.00809
MPT-2	-0.17790	-0.01234

S3. Transient Fluorescence Lifetime Analysis

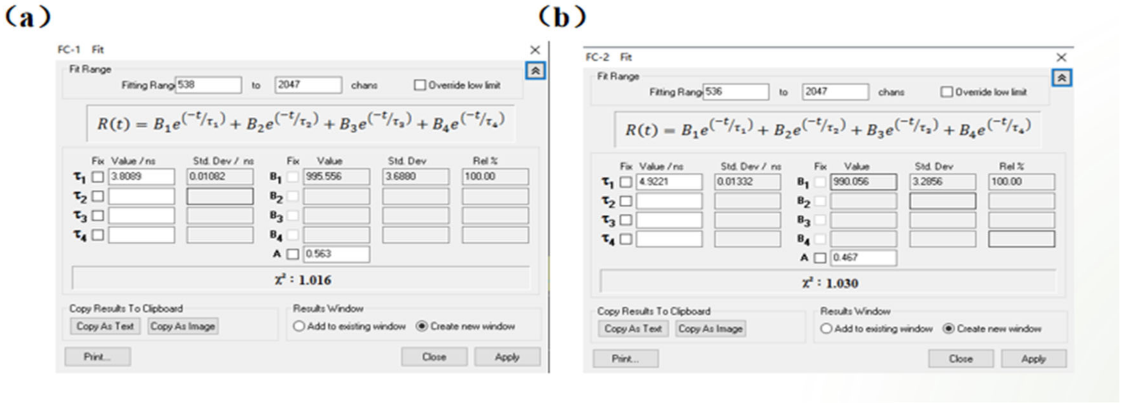

Figure S2. (a) and (b) are the fitting results of TCSPC, with reduced chi-square values of 1.016 and 1.030, respectively, indicating that the single-exponential model fits well. The uncertainties shown are the standard errors from the least squares fitting, mainly arising from photon counting statistics, with a relative uncertainty of about 0.3%, excluding systematic errors such as IRF convolution and time calibration. It is specifically noted that the four-exponential model used in the software is only for initial screening, and the other components are not used in the actual fitting. In addition, the IRF width of the FLS1000 is much smaller than the measured lifetime (3.80 ns and 4.92 ns), contributing very little to systematic error. The uncertainty on the order of tens of picoseconds mainly arises from photon counting statistics and is within the normal range for nanosecond-level lifetime measurements.

S4. Stokes Shift

The apparent Stokes shift is defined as the wavelength difference between the lowest energy absorption wavelength and the maximum emission wavelength:

$$Stokes\ shift = \lambda_{em} - \lambda_{abs} \qquad S(2)$$

It should be noted that this value incorporates multiple contributions—vibrational relaxation, solvent relaxation, and excited-state conformational relaxation—and therefore does not correspond to the strict 0–0 Stokes shift; it is instead used solely for relative comparisons among compounds within the same series under identical experimental conditions.

S5. Material NMR Test Results

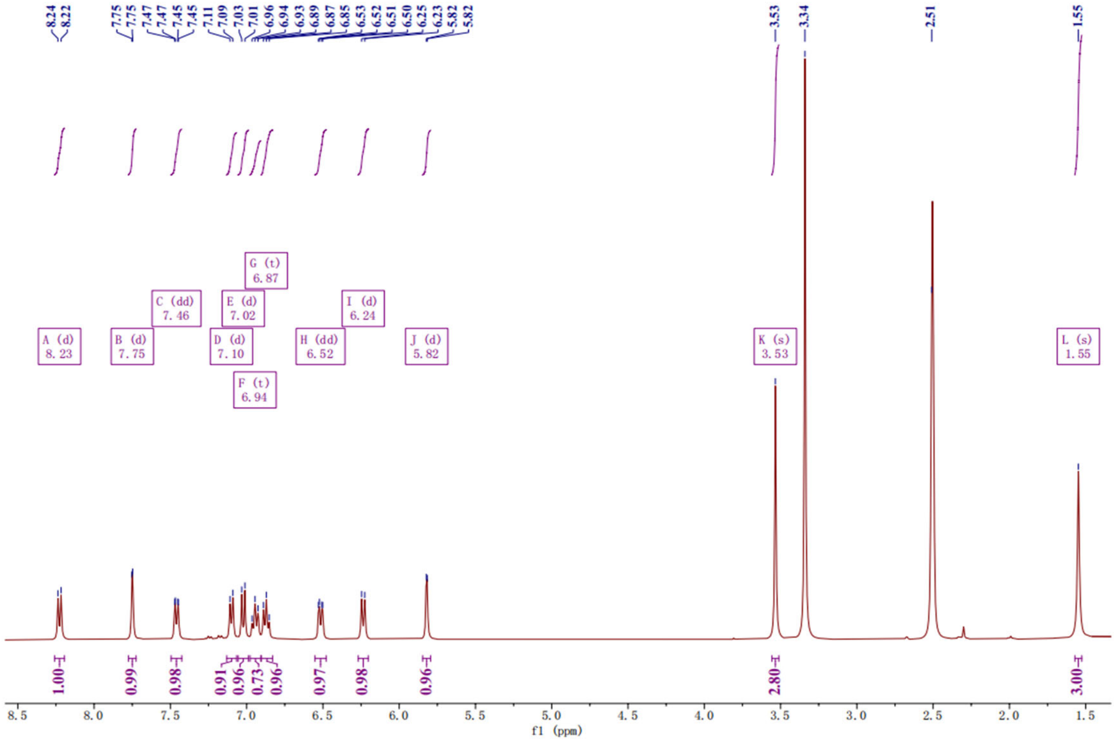

Figure S3. ^{1}H NMR spectrum of MPT-1 in DMSO.

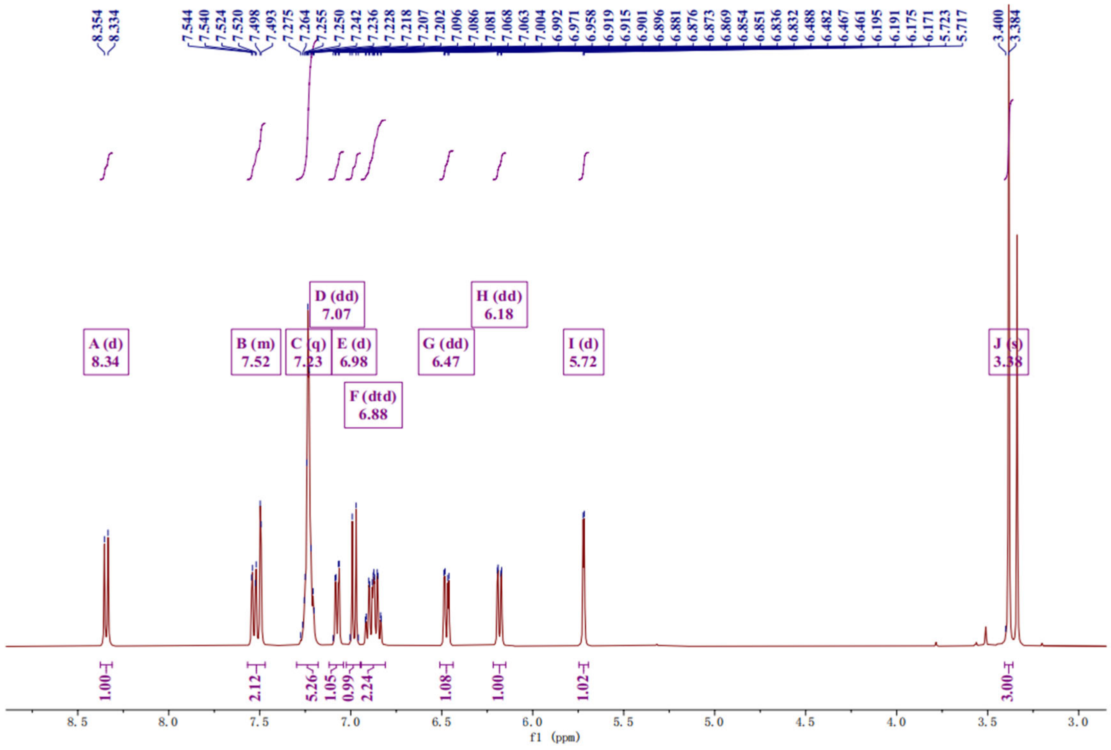

Figure S4. ^{1}H NMR spectrum of MPT-2 in DMSO.

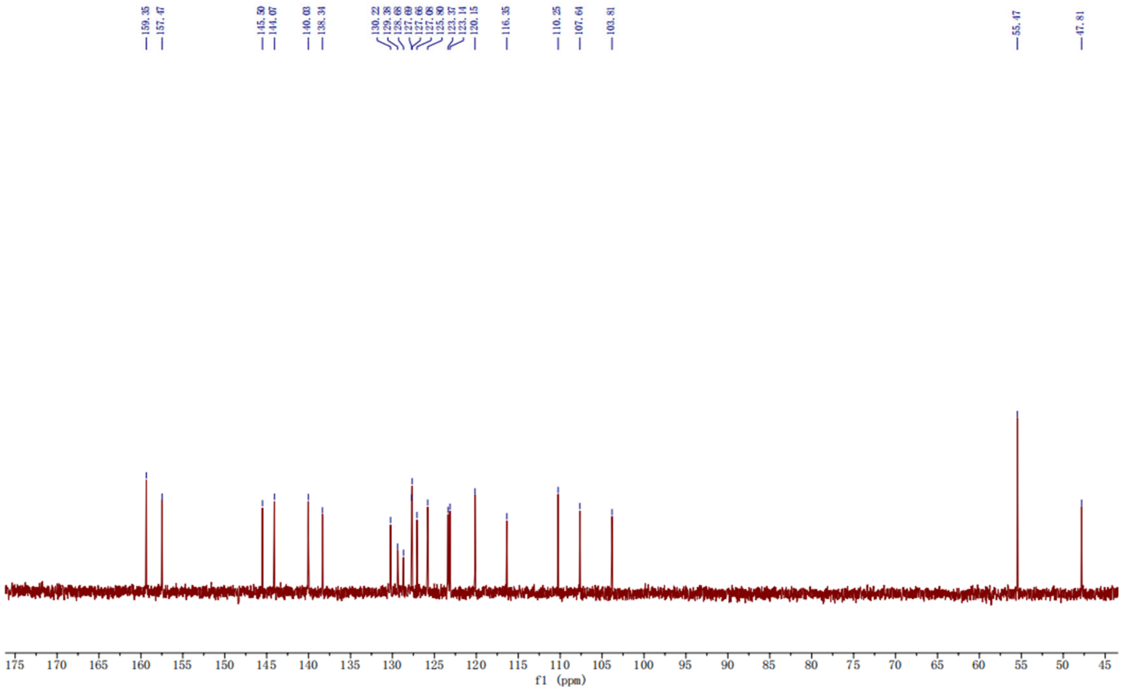

Figure S5. ^{13}C NMR spectrum of MPT-1 in DMSO.

:

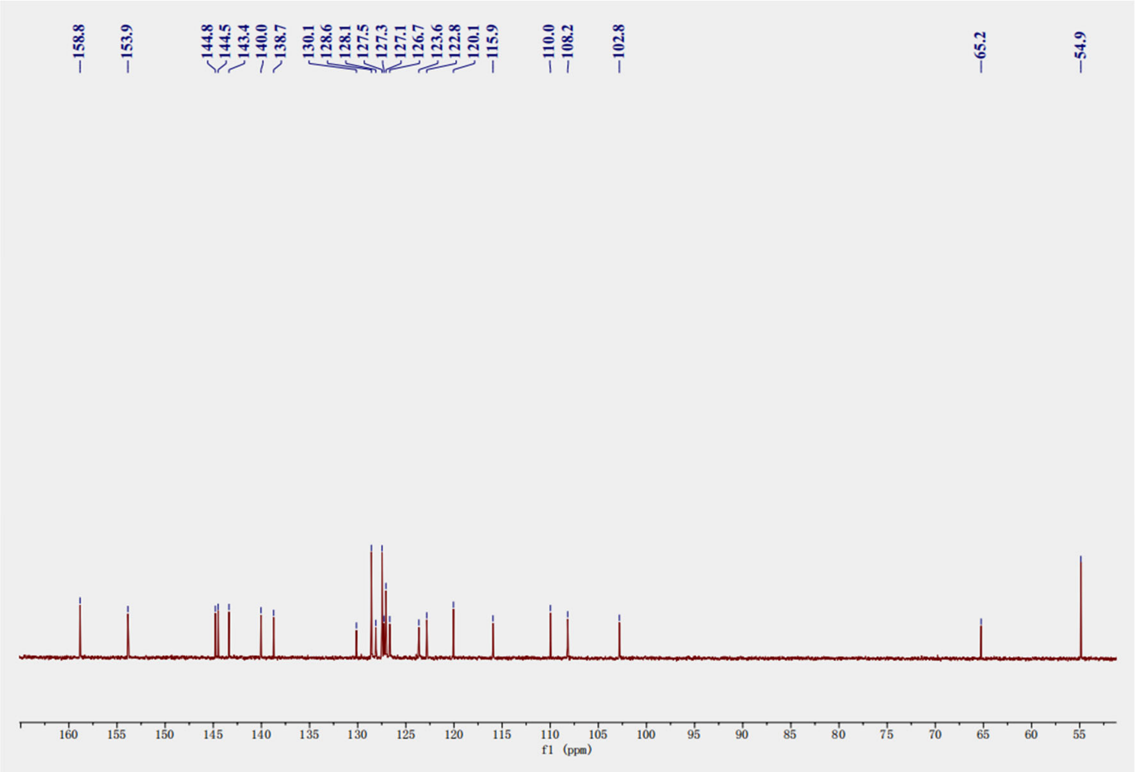

Figure S6. ^{13}C NMR spectrum of MPT-2 in DMSO.

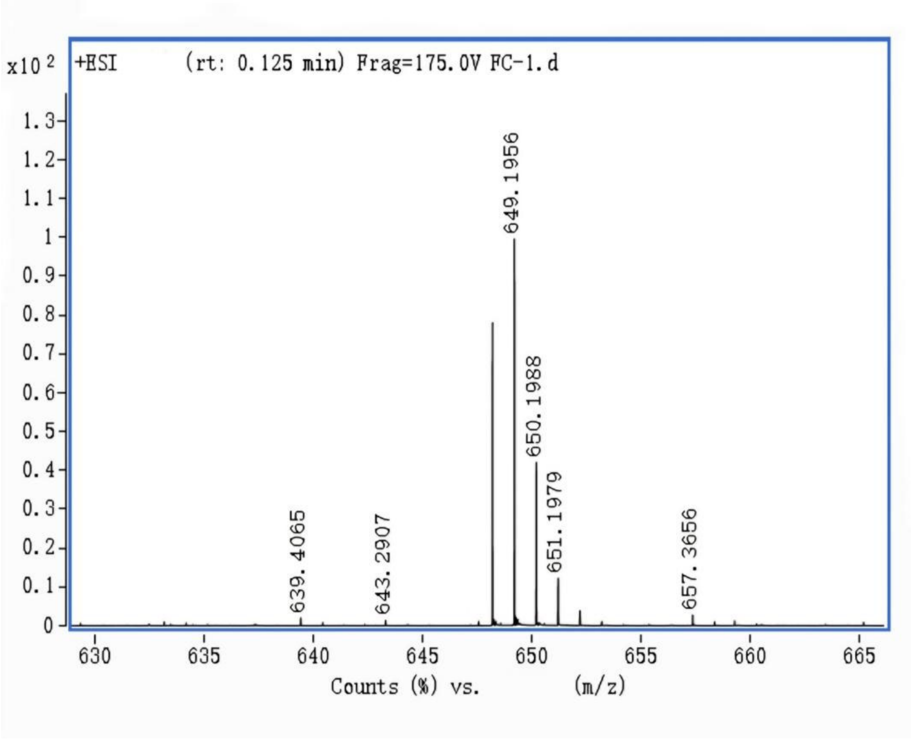

Figure S7. HR-MS spectrum of MPT-1 (margin of error (< 5 ppm)).

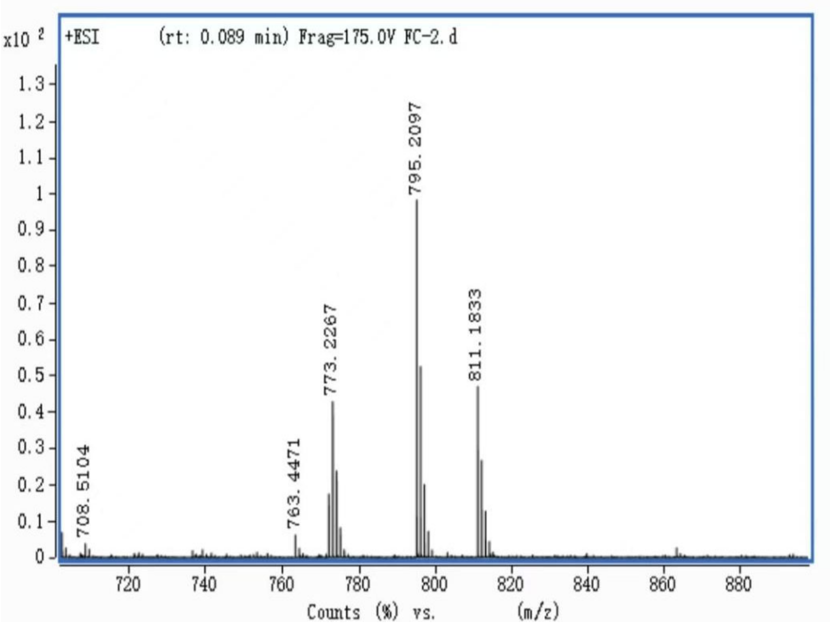

Figure S8. HR-MS spectrum of MPT-2 (margin of error (< 5 ppm)).

S6. PLQY Integrating Sphere Methodology and Uncertainties

(1) Solution PLQY Measurement (Absolute Integrating Sphere Method; equipment is Edinburgh FS5)

Place the liquid sample in a quartz cuvette and use the pure solvent (chloroform) as a blank reference. Using a fluorescence spectrophotometer equipped with an integrating sphere, fix the excitation wavelength. First, measure the blank sample and record the integrals in the excitation scatter region and the emission region; then measure the sample under the same conditions and record the corresponding integrals in the excitation scatter region and emission region. Finally, calculate the fluorescence quantum yield using the instrument software. The scattering integral ranges for MPT-1 and MPT-2 are 400-420 nm and 410–433 nm respectively, and the emission integral ranges are 468-599 nm and 460-649 nm, with absorbances of 0.4 and 0.3 respectively. Blank samples are measured under the same conditions. Although these absorbances are higher than the values recommended for the relative method, the integrating sphere method accounts for the effect of absorbance by directly measuring the number of absorbed and emitted photons in the calculation.

(2) Solid-State PLQY Measurement (Absolute Integrating Sphere Method; equipment is Edinburgh FS5)

Add the solid sample to the solid sample holder and flatten it for fluorescence spectrum acquisition. Prepare a corresponding blank sample holder to subtract the background. Calculate the fluorescence quantum yield using the instrument software. The scattering integral range for MPT-1 is 400–420 nm and the emission integral range is 468–599 nm, with an absorbance of 0.40; the scattering integral range for MPT-2 is 410–430 nm and the emission integral range is 460-649 nm, with an absorbance of 0.30. The uncertainty in solid-state PLQY measurements mainly arises from the roughness of the powder sample and reabsorption effects.
